# Machine learning and statistics shape a novel path in archaeal promoter annotation

**DOI:** 10.1186/s12859-022-04714-x

**Published:** 2022-05-10

**Authors:** Gustavo Sganzerla Martinez, Ernesto Pérez-Rueda, Sharmilee Sarkar, Aditya Kumar, Scheila de Ávila e Silva

**Affiliations:** 1 Programa de Pós-Graduação em Biotecnologia, Universidade de Caxias do Sul, Av. Francisco Getúlio Vargas, 1130, Caxias do Sul, RS CEP 95070-560 Brazil; 2 Department of Molecular Biology and Biotechnology, Tezpur University, Tezpur, Assam 784028 India; 3 Instituto de Investigaciones en Matemáticas Aplicadas y en Sistemas, Universidad Nacional Autónoma de México, Unidad Académica de Yucatán, Yucatán Mérida, Mexico

## Abstract

**Background:**

Archaea are a vast and unexplored domain. Bioinformatic techniques might enlighten the path to a higher quality genome annotation in varied organisms. Promoter sequences of archaea have the action of a plethora of proteins upon it. The conservation found in a structural level of the binding site of proteins such as TBP, TFB, and TFE aids RNAP-DNA stabilization and makes the archaeal promoter prone to be explored by statistical and machine learning techniques.

**Results and discussions:**

In this study, experimentally verified promoter sequences of the organisms *Haloferax volcanii*, *Sulfolobus solfataricus*, and *Thermococcus kodakarensis* were converted into DNA duplex stability attributes (*i.e.* numerical variables) and were classified through Artificial Neural Networks and an in-house statistical method of classification, being tested with three forms of controls. The recognition of these promoters enabled its use to validate unannotated promoter sequences in other organisms. As a result, the binding site of basal transcription factors was located through a DNA duplex stability codification. Additionally, the classification presented satisfactory results (above 90%) among varied levels of control.

**Concluding remarks:**

The classification models were employed to perform genomic annotation into the archaea *Aciduliprofundum boonei* and *Thermofilum pendens*, from which potential promoters have been identified and uploaded into public repositories.

**Supplementary Information:**

The online version contains supplementary material available at 10.1186/s12859-022-04714-x.

## Background

The righteous introduction of the archaeal domain to the tree of life dates no longer than half a century. Since then, a lush path towards discovering new insights in order to benefit archaeal genome annotation arose. The archaeal domain is diverse [1, 2], ranging from Earth's most extreme environments to our guts. Hence, finding a model organism that represents the whole expanse of this domain is rather a simple-minded and reductionist task. At least 13 families in the archaea phylogenetic tree might be spotted, which have huge dissimilarities both in their genetic and phenotype setting [3, 4], as well as elements that orchestrate the cell necessities.

Single cell organisms rely on finely regulated cellular processes. The production of the right nutrient at the right moment grants the cell survivability. Instances of these processes include the transcription of an RNA molecule. This mid-step operation is carried out by the RNAP enzyme and configures a central process in the genetic information flux across all domains. The way the transcription occurs in archaea roughly resembles the eukaryotes [5]. In fact, these two domains are evolutionary siblings and archaea might have given origin to eukarya [6]. The overall structure of this process in these two domains presents a certain level of conservation. Indeed, the eukaryotic model poses as a more specialized version of its archaeal counterpart. For instance, while archaea employs a single RNAP to transcribe all genes, animals and plants make use of three and five different enzymes, respectively [7, 8].

The recruitment of RNAP to the DNA is mediated by a DNA segment defined as a promoter sequence whose presence is necessary for the initiation of the transcription. The typical archaeal promoter element possesses three basal transcription factor binding sites. These additional proteins are TATA-box Binding Protein (TBP), Transcription Factor B (TFB), and Transcription Factor E (TFE) and they are needed for correctly directing RNAP to its precise site of action [9]. On a nucleic acid level, these proteins bind to: (*i*) a wTTATwww set of nucleotides, located at − 25, matching the TBP binding site, where w means A or T in the IUPAC code; (*ii*) an ssnAA sequence located around two nucleotides upstream TATA and a TAC sequence located in the range of − 1/ − 10, due to its two-extremity binding, TFB stabilizes TBP and the two combined create the Pre Initiation Complex (PIC), where s means C or G and n means any nucleotide in the IUPAC code; (*iii*) a TFE protein has the function of assisting PIC formation, hence, its binding preference varies according to the promoter and organism [9, 10].

The conservation found around the binding site of transcription factor proteins in the archaeal genome might be used as input in a way that the recognition of these regulators is able to provide a more reliable annotation. The promoter prediction task is well developed in other branches of life than archaea. Such tools have succeeded in classifying these regulators in eukarya and bacteria. However, due to the particularities archaea have, a universal promoter classifier is an open scientific question.

In this work, we systematically locate the potential promoters of unannotated archaea by using their structural properties in comparison to random and sequences where no promoters have been identified. To do so, the well-conserved nature of archaeal promoters is employed and stressed.

## Materials and methods

### Promoter sequences

A total of 3630 experimentally validated promoters of three different archaea were employed in this study: 1340 sequences of *Haloferax volcanii*, 1048 sequences of *Sulfolobus solfataricus*, and 1248 sequences of *Thermococcus kodakarensis*. These are model organisms in the Euryarchaea and TACK superphylum. These organisms were selected because there is available transcriptome information, which enables the extraction of promoter sequences associated with a given transcript.

The original data contains 1001 nucleotides per sequence with their Transcription Start Sites (TSS) mapped. Only primary TSS (pTSS) from the published transcriptomic data was considered. Next, a sub-sequence containing 100 nucleotides, i.e. − 80 to + 20 was extracted. This region comprises the reported core promoter in *H. volcanii* and *S. solfataricus* [11, 12] and it has been reported as sufficient to initiate transcription in archaea [12]. Furthermore, the precise location of these organisms' promoters was reported to be located in the proposed range [12–16]. Annotations and lists of the promoters used in this study are available at https://zenodo.org/record/5137551.

### Control datasets

The classification methods of this study were stressed with three forms of control. First, we, through a self-developed Python script, shuffled the 100-nucleotides original sequences. A second control dataset was used by selecting the downstream sequences from + 21 to + 121. By this, we wanted to test the validity of our method by assessing sequences that do not indicate promoter activity nor have a TATA-box; and finally, we performed a second method for shuffling sequences, proposed by [17]; i.e., we divided the 100 nucleotide sequences into 20 blocks of 5 nucleotides each, then, we shuffled 12 of the blocks. By doing this, the consensual motifs such as TATA-boxes might be preserved in a way that our identification method is tensioned.

### Structural parametrization

The totality of the sequences of this study (promoters and controls) were submitted through a structural coding in order to represent genetic information into numeric attributes. This representation captures specific sequence properties associated with regulatory regions such as promoters [18]. The parameter chosen for this study is DNA Duplex Stability (DDS). This feature has been employed as a way to represent the richness of GC base-pairs due to their extra hydrogen bond [19–23]. In this regard, Eq. 1 was used to calculate the DNA duplexes reported in [21]. It hinges on the assignment of a numeric attribute in sliding dinucleotide windows.

1 $$G = {\Delta }_{i,i + 1}^{0}$$

### Classification through a statistical approach

Firstly, position-specific slices of 8 nucleotides (6 nucleotides matching the TATA-box, a spacer of 2 nucleotides, and 2 nucleotides comprising the BRE element) were extracted and averaged in each dataset (promoters, and three controls). Then, an interval was set ranging from the plus and minus values of the standard deviation formed upon the promoter dataset (Eq. 2).

2 $$Interval = \overline{x}_{promoter} \pm \sigma_{promoter}$$

Finally, a sequence was labeled as a promoter if its TATA + BRE nucleotides belongs to the range of $Interval$. Otherwise, it was classified as a non-promoter. A visual representation of the statistical method for classifying archaeal promoter sequences is available at: https://doi.org/10.5281/zenodo.5154110.

### Classification through an artificial neural network approach

In order to validate the simulation process, a *k*-fold-cross validation method was considered, where *k* = 10. This method involves in reserving 1/10 of the dataset to be used in the testing. The training is done with the remaining 9/10 shares. This grants that any biased data point gets covered. The validation process was done following the *sample* method in R [24].

Artificial Neural Network (ANN) simulations took place in the R environment through the *neuralnet* package [25]. The algorithm chosen to fit the ANN was the resilient backpropagation, since it has already succeeded in classifying genomic data [26]. The number of neurons in the hidden layer was set to 2 since a too complex curve to fit data points might be seen as a non-productive decision in machine learning [27]. The number of iterations over the training dataset, i.e. epochs, was increased until the validation and training errors kept dropping [28]. Finally, the maximum number of steps the ANN was allowed to reach until convergence was 200,000 as an attempt to balance computational costs. The R script that performed the ANN simulation is available at https://github.com/gustavsganzerla/ANN---Archaeal-classification.git.

### Assessment of classification

A binary classification might get assessed through an error matrix with predicted and actual values of a classification; this enables the performance of a classification technique to be evaluated. The elements that compose the matrix belong to two classes, positives and negatives and they are: True Positives (TPs), which correspond to the number of elements of a *d* class correctly predicted as a member of *d* class; True Negatives (TNs), which are elements that do not belong to a *d* class and have been assigned as non-*d* class; False positives (FPs), which comprises a member of *d* class classified as a non-*d* class; and False negatives (FNs), which encompasses members that do not belong to a *d* class and have been assigned as *d* class. With the error matrix, it is possible to calculate performance metrics of a binary classification.

The first metric is Accuracy (Eq. 3), which measures the proportion of correct predictions in the whole dataset (both TPs and TNs).

3 $$Accuracy = \frac{(TP + TN}{{\left( {TP + TN + FP + FN} \right)}}$$

The second metric is Precision that verifies how many of the observations predicted as positive are actually positive and it is calculated through Eq. 4.

4 $$Precision = \frac{TP}{{\left( {TP + FP} \right)}}$$

Next, Recall, which assesses how many of the TPs are actual TPs, is obtained through Eq. 5.

5 $$Recall = \frac{TP}{{\left( {TP + FN} \right)}}$$

Finally, Specificity that calculates the detection rate of TNs throughout the entire dataset. It is obtained by Eq. 6.

6 $$Specificity = \frac{TN}{{\left( {TN + FP} \right)}}$$

### Validation of the methods

**Fig. 1:**
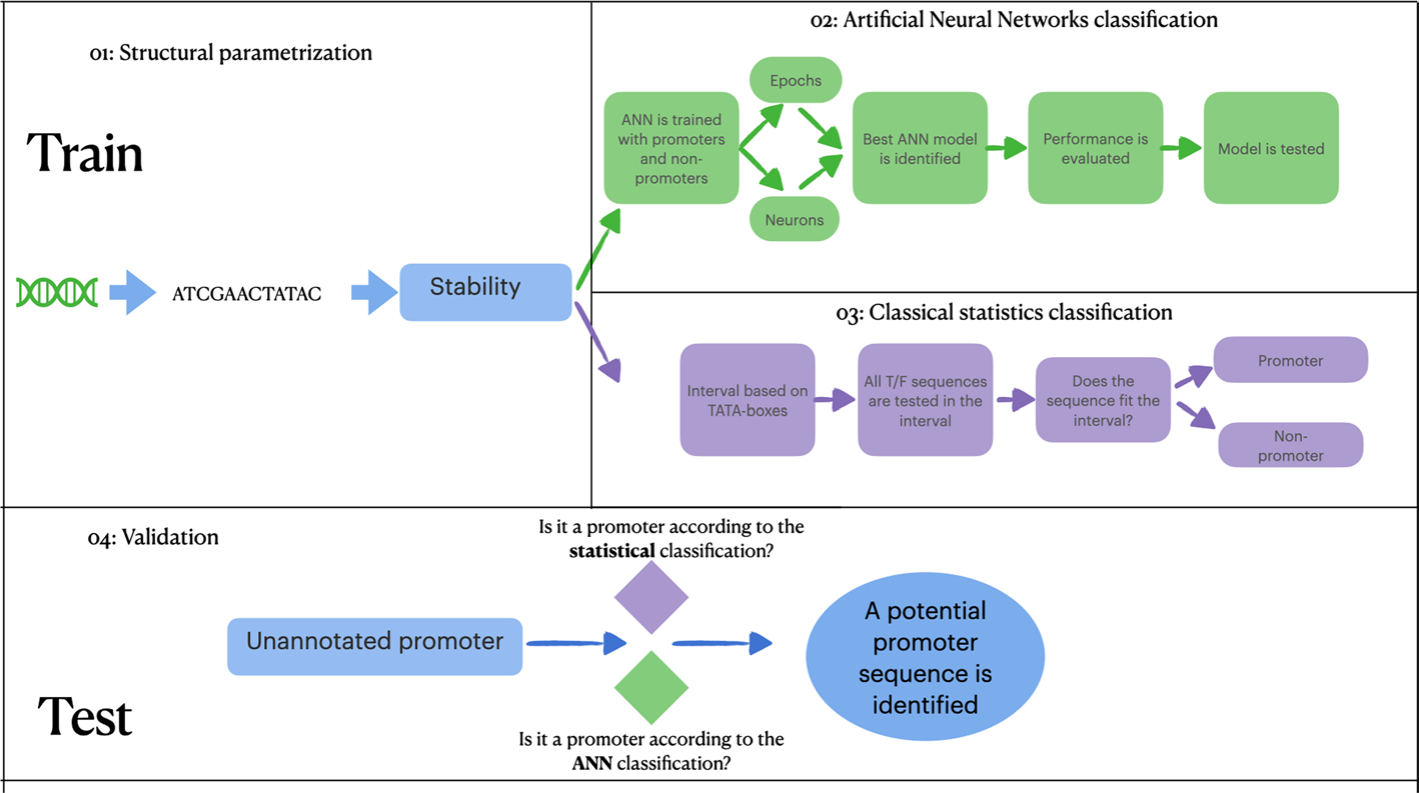
Overview of the classification rationale employed in this study. The figure is divided into i, ii, iii (train) and iv (test). (i) represents the conversion of genetic information into numeric attributes related to DDS, which is used as input of two classification methods. (ii) matches the Artificial Neural Network phase of classification; (iii) conveys information of how the classification was achieved through statistics. Both ii and iii were performed with experimentally verified promoters. Finally, the test, (iv) represents the validation process with upstream sequences whose promoters have not been identified yet. Each sequence undergoes through i, ii, and iii; then, the final decision is computed whether the sequence is a promoter or not

To provide a validation for the methods proposed in this study, upstream sequences whose promoter activity has not been experimentally described yet, were downloaded from the RSAT prokaryotic database (http://embnet.ccg.unam.mx/rsat/) in its Sep 23 12:30:06 2021 version. The database contains upstream regions for 211 archaeal organisms. Two archaeal genomes, *Aciduliprofundum boonei* (741 sequences of 400 nucleotides each) and *Thermofilum pendens* (1926 sequences of 400 nucleotides each) exhibited a promoter-like profile in a previous study [29], i.e. the codification into DDS of upstream regions was found to be statistically similar to experimentally validated promoters, indicating that these particular upstream regions might contain promoters. Therefore, 400 nucleotides sequences got their TATA-box and TFB sites extracted. The nonparametric Kruskal Wallis test was employed to determine if the groups of experimental and potential promoters hold statistical differences. Finally, lists of annotated potential promoters of these two organisms are provided. A flowchart describing the classification method and the validation of the findings is described in Fig. 1.

## Results and discussions

### DNA duplex stability parametrized archaeal promoters differ from control sequences

**Fig. 2:**
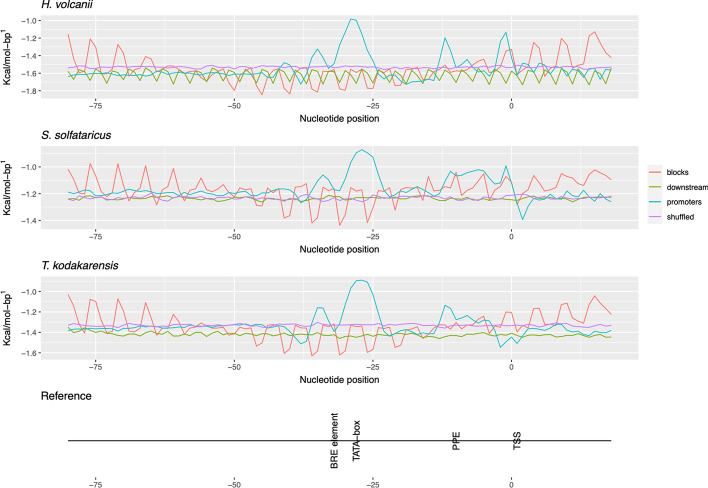
Signal comparison between promoters and controls in three archaea. The core promoter region (− 80 to + 20) of three archaea were extracted and converted into DNA Duplex Stability. The three archaea explored in this study have each a separate panel (*H. volcanii*, *S. solfataricus*, and *T. kodakarensis).* Moreover, a reference panel (Reference) is provided, in which the binding site of the proteins TBP (TATA-box), TFB (BRE element), and TFE (proximal promoter element) are depicted. The peaks in DDS of the three organisms match the binding sites of the aforementioned proteins

In order for getting the binding sites of transcription factor proteins represented by numeric inputs, genetic information was coded into DDS. Promoter sequences have already been well represented by DDS [19]. Concerning the coding of genetic information into DDS as well as locating areas of interest for turning promoters unmatched, Fig. 2 has been provided. The plotting of promoter sequences and their negative controls reveal that the binding site of transcription factor proteins is only found within promoters; these areas are observed in the promoter line, around positions − 28, − 32, and in the range of − 10 to + 1.

The initiation of transcription in archaea has been reported to need two transcription factor proteins: a TBP and a TFB, homolog to eukaryotic TFIIB [7, 10]. Additionally, a second strong signal was observed around positions − 10 and + 1, matching the Proximal Promoter Element. This area consists of the binding site of a protein namely TFE, which has been reported to optimize the transcription in archaea by stabilizing the formation of a PIC [30]. Considering these organisms have limited genomes and need to have their metabolic demands matched in order to thrive, the presence of transcription optimizer proteins such as TFE plays a pivotal role in the gene expression.

Next, we have observed conserved binding sites of promoter recruitment transcription factor proteins in archaea with varied GC content (*H. volcanii* = 66.13%, *T. kodakarensis* = 50.67%, and *S. solfataricus* = 34.48%), More GC would indicate less potential binding sites for such proteins, as reported in [29]. However, our rationale has been able to find the binding site despite the amount of GC in a particular archaeon, Therefore, the binding site of these three proteins are clear in the plots representing the promoters in all organisms, suggesting that DDS succeeded in well representing promoter elements in archaea.

### Statistical classification succeeds in the distinction of promoter sequences

In order to promote a classification method, the mean values of TATA + BRE sites of promoter sequences as well as three levels of control were converted into DDS. For each observation, the average of the promoter sequence differs from the three levels of control, where the closer from the promoter score is the shuffled sequence, followed by the downstream, and finally, the block shuffling process (Table 1).

**Table 1:** Flagships of archaeal classification based on statistics

	Promoters	Standard deviation	Blocks	Downstream	Shuffled
*H. volcanii*	− 8.36	± 1.15	− 11.66	− 11.44	− 10.66
*S. solfataricus*	− 6.72	± 0.85	− 8.87	− 8.58	− 8.73
*T. kodakarensis*	− 7.13	± 0.93	− 10.16	− 10.07	− 9.28

The results displayed in Table 1 have information on the averaged values of the region encompassing TBP and TFB (BRE extremity) for four datasets: (*i*) experimentally verified promoters; (*ii*) control done in blocks; (*iii*) downstream sequences as control and; (*iv*) control with shuffled sequences. The standard deviation for the average of the promoters is also provided

The dissimilarity observed in the promoters and the three forms of controls in Table 1 enables the statistical form of classification proposed in this study. The promoter interval was ranged and all the sequences got their data classified into promoter or non-promoter. The results were then computed on an error matrix (Table 2), from which the precision value remains the same in every organism against their controls, since its calculation relies on positive values. The assessment of Table 2 indicates a higher recall value. The most satisfactory scores were achieved by the block form of control whilst the last was found in shuffled sequences, with the exception of *S. solfataricus*, in which downstream drags behind shuffled.

**Table 2:** Results of the statistical method of classification

		Accuracy (%)	Precision (%)	Recall (%)	Specificity (%)
*H. volcanii*	Blocks	79.13	67.81	87.64	73.76
	Downstream	78.76	67.81	86.8	73.6
	Shuffled	72.04	67.81	74.06	70.33
*S. solfataricus*	Blocks	78.55	77.44	79.19	77.93
	Downstream	78.22	77.44	78.65	77.8
	Shuffled	78.55	77.44	79.19	77.93
*T. kodakarensis*	Blocks	81.37	70.11	90.48	75.6
	Downstream	81.16	70.11	90.02	75.52
	Shuffled	74.63	70.11	77.09	72.59

Performance metrics derived from a confusion matrix. The precision value was found the same in each organism due to the first class (promoters) not changing with new forms of control

The statistical method of classification reported in this study has proven satisfactory in a way that it did not employ techniques encompassing machine learning. Firstly, the lower Precision value in the method suggests the model classified too many False Positives, this means non-promoters were classified as promoters in some instances. A reason for this to happen is the diversification found in archaea [2] and the dissimilarity in owning conserved binding sites [29]. Secondly, the most fine counts were achieved in the block form of control, matching the identification of Table 1, in which the means of the blocks are the furthest from the promoters. Additionally, the method has presented satisfactory scores regarding recall, a metric that is sensible towards False Negatives. This phenomenon is explained due to the transcription machinery of different archaea being quite similar. If a sequence that lacks conserved binding sites of TBP and TFB, it is very unlikely to be classified as an archaeal promoter.

The stress of the statistical model, brought by an inter-archaea classification (Table 3), similarities have been found in *S. solfataricus* and *T. kodakarensis*, confirming what was proposed by Takemasa et al. [31] in order to turn *T. kodakarensis* and *S. solfataricus* as regulatory chassis for hyperthermophilic archaea. These two particular organisms were reported to be similar in terms of their AT% throughout the genome [29], while *H. volcanii* has higher GC. We found the nucleotide composition directly affects the classification outcome, since it relies on conserved binding sites of transcription factor proteins.

**Table 3:** Results of the inter-organism statistical method of classification

		*H. volcanii*	*S. solfataricus*	*T. kodakarensis*
*H. volcanii*	Accuracy (%)	–	61.68	68.92
	Precision (%)	–	24.7	40.89
	Recall (%)	–	90.05	93.34
	Specificity (%)	–	56.71	62.11
*S. solfataricus*	Accuracy (%)	32.17	–	78.44
	Precision (%)	21.78	–	77.44
	Recall (%)	27.51	–	79.01
	Specificity (%)	35.23	–	77.88
*T. kodakarensis*	Accuracy (%)	50.47	82.9	–
	Precision (%)	40.62	72.43	–
	Recall (%)	50.99	91.86	–
	Specificity (%)	50.21	77.17	–

The classification rationale (formed upon the interval of mean $\pm$ standard deviation) was tested in different organisms. The results displayed in each cell have information of the averaged results of the three forms of control. The test of an archaeon with its own rationale for statistical classification was omitted, for it, see Table 2

Statistics has been proved as an adequate way to classify promoter sequences of archaea. This method is highlighted to its ease to implement, since it does not require extensive computational costs. Indeed, descriptive statistics is seen as a precursor of machine learning in classificatory nature [32].

### Artificial Neural Network conveys a sturdier classification

**Fig. 3:**
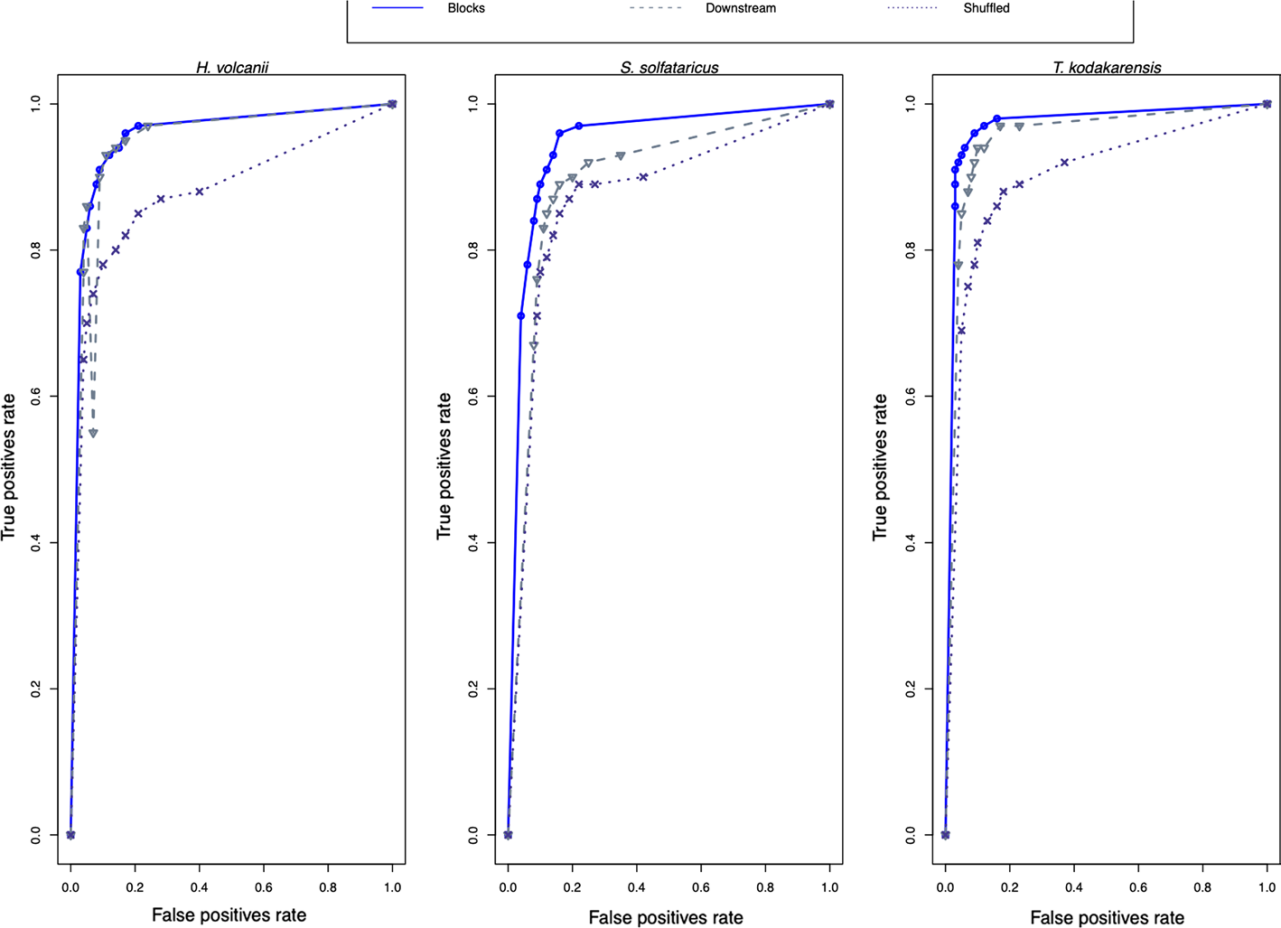
ROC curves for the best ANN simulation in each archaeon. Once the best architecture for classifying archaea (each organism is presented in a separate panel) with neural networks was defined, the classification threshold was adjusted to produce ROC curves. The default output neuron yields a value and if it's bigger than 0.5, it gets classified as a promoter, otherwise, it is classified as a non-promoter. Each tick in the ROC curves represents an adjusted decision threshold, varying from 0 (x axis = 0, y axis = 0) to 1 (x axis = 1, y axis = 1)

In order to achieve more robust classification scores, ANNs were used. In the ANN simulation, the architecture that protruded satisfactory scores follows: (*i*) seven neurons in the input layer; (*ii*) two neurons in the hidden layer, and; (*iii*) one neuron in the output layer. Table 4 indicates the results achieved by the ANN simulation, with a default tradeoff value of 0.5 in computing the output of the model. The four parameters tested in the classification (Accuracy, Precision, Recall, and Specificity) are evenly spread among different forms of control. For a mean of the three forms of control against each classification parameter, please see Additional file 1: Table S1, in which the results of the four metrics are equidistant. Furthermore, the behavior of the ANN model was tested with different tradeoff values through a ROC (Receiver Operator Characteristic) curve, presented in Fig. 3.

**Table 4:** Results of the ANN-based classification

		Accuracy (%)	Precision (%)	Recall (%)	Specificity (%)
*H. volcanii*	Blocks	92.48	93.05	92.03	92.96
	Downstream	91.08	90.67	91.45	90.77
	Shuffled	84.55	84.86	84.27	84.94
*S. solfataricus*	Blocks	89.03	91.43	87.01	91.18
	Downstream	87.36	86.93	88.23	86.48
	Shuffled	86.63	84.56	88.27	85.17
*T. kodakarensis*	Blocks	94.96	93.39	96.21	93.83
	Downstream	91.35	91.69	91.31	91.46
	Shuffled	86.46	84.1	89.12	84.36

Each cell of this table contains the performance achieved by the best epoch for weight updating across the training dataset, i. e. the epochs were no longer increased when the convergence error became stable. For more details on the ANN simulation, see "Classification through an artificial neural network approach" and "Artificial Neural Network conveys a sturdier classification" sections

A second application of ANNs was conducted to evaluate if the pattern of one archaeon might be employed to classify another. Following this rationale, a new simulation was achieved in which the ANN was trained with one organism and tested with another. The results of this new simulation are available in Table 5, from which there is a leaning towards *S. solfataricus* and *T. kodakarensis*. The *H. volcanii* logistics produced classification results far distant from each other (e.g. from 60.87% recall to 96.23% specificity in a crossing of *S. solfataricus* and *H. volcanii*).

**Table 5:** Results of the inter-organism ANN method of classification

		*H. volcanii*	*S. solfataricus*	*T. kodakarensis*
*H. volcanii*	Accuracy (%)	–	70.63	81.61
	Precision (%)	–	41.75	66.56
	Recall (%)	–	95.95	94.68
	Specificity (%)	–	63.89	74.72
*S. solfataricus*	Accuracy (%)	66.51	–	86.46
	Precision (%)	98.89	–	95.45
	Recall (%)	60.87	–	81.04
	Specificity (%)	96.23	–	94.42
*T. kodakarensis*	Accuracy (%)	75.42	82.48	–
	Precision (%)	97.52	71.56	–
	Recall (%)	68.9	91.48	–
	Specificity (%)	94.34	77.01	–

In this classification, the architecture that was trained with data of one archaeon and its controls was tested with other organisms and their controls. The results displayed in each cell have information of the averaged results of the three forms of control. The testing data of an archaeon with its own ANN architecture was omitted, for it, see Table 3

The results brought by the ANN classification suggest the model succeeded in classifying archaeal promoters, distinguishing them from three variations of control. In fact, this machine learning approach has succeeded in encountering promoters [17, 22, 23]. An implementation of similar nature was performed in [23] through the classification of bacterial promoters. The results obtained in this present study outperformed the bacterial classification because of the structure of the archaeal promoter in comparison to bacteria, which contains sigma factor proteins to direct RNAP to specific sites.

By outshining the statistical classification, the mathematical robustness of the ANN method [33] has proven uneven. Also, the rationale found in such method has matched the statistical classification, but overcame it. A good indicator to observe prediction validity is brought by ROC curves, which plots the specificity cost in gaining more recall [34]. The most evident characteristics are observed in the block control, which is found in the upper left corner of the plotting areas, confirming what the statistical analysis has found and validating the findings of Table 4 and Additional file 1: Table S1. The evenly spread scores (not fluctuating more than 1% in the metrics of each archaeon) certify the success of classification of ANN, suggesting the conservation protruded by a DDS codification of transcription factor binding sites has sufficiently turned promoter sequences unique.

The verification of inter-organism rationale of classification has evidenced that *S. solfataricus* and *T. kodakarensis* share similarities, evidenced by the acceptable classification scores between these two archaea. The high values of recall observed in *H. volcanii* vs. *S. solfataricus* and *T. kodakarensis* suggest that very few False Positives were identified, meaning that it was rare for the model to incorrectly classify *H. volcanii* promoters, this is due to the divergent amount of GC in this organism, reported in [29]. The bumpy results of precision in *H. volcanii* and *S. solfataricus* (and vice-versa) shows that the *S. solfataricus* model correctly identified non-promoters of the *H. volcanii* dataset, meaning the model correctly identifies promoters with conserved binding sites. However, the *H. volcanii* ANN architecture failed in classifying non promoters of the *S. solfataricus* dataset, indicating that the rationale of classification of this halophilic archaeon only performs well with organisms with higher GC%. In general terms, due to the higher amount of GC in *H. volcanii* and consequently, less conserved binding sites of transcription factors, the promoter sequences of this organism are unparalleled.

### ANNs and statistics employed in finding potential archaeal promoters

Upstream regions of *Aciduliprofundum boonei* and *Thermofilum pendens* were selected in order to extract potential promoters from. The statistical and ANN models found in *S. solfataricus* and *T. kodakarensis* were employed in the validation dataset. *H. volcanii* was left out due to its unparalleled AT content; such inclusion would have jeopardized the validation. An upstream region was considered as a promoter if the statistics of *S. solfataricus* and *T. kodakarensis* and the ANN of *S. solfataricus* and *T. kodakarensis* flagged the given sequence as a promoter. From the 742 and 1927 sequences from *A. boonei* and *T. pendens*, respectively, the method encountered 145 promoters of the Euryarchaea and 243 promoters of the Crenarchaea. The lists containing sequence ID, the nucleotide sequences, and functional annotation are available at https://doi.org/10.5281/zenodo.5729308.

**Fig. 4:**
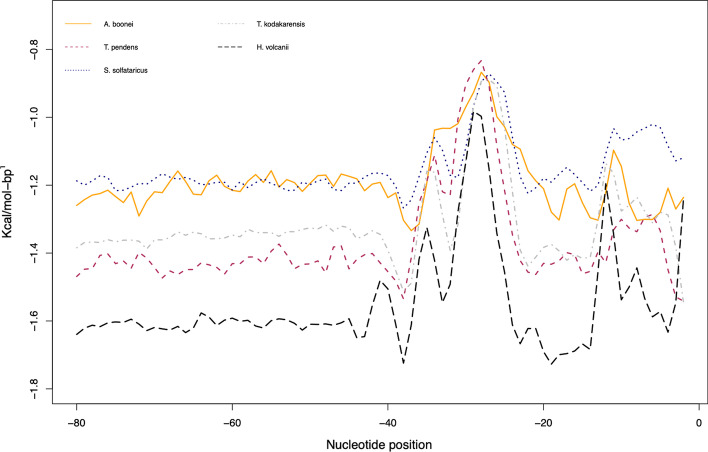
Comparison of physical profiles in annotated *vs* unannotated archaea. A promoter segment (− 80 to 0) was extracted from the organisms: *H. volcanii*, *S. solfataricus*, *T. kodakarensis*, *A. boonei*, and *T. pendens* and converted into DNA Duplex Stability. The last two are derived upon upstream sequences of the organisms, hence, no annonnation regarding promoters is available. The observed peaks of the validated promoters match the unannotated ones

**Fig. 5:**
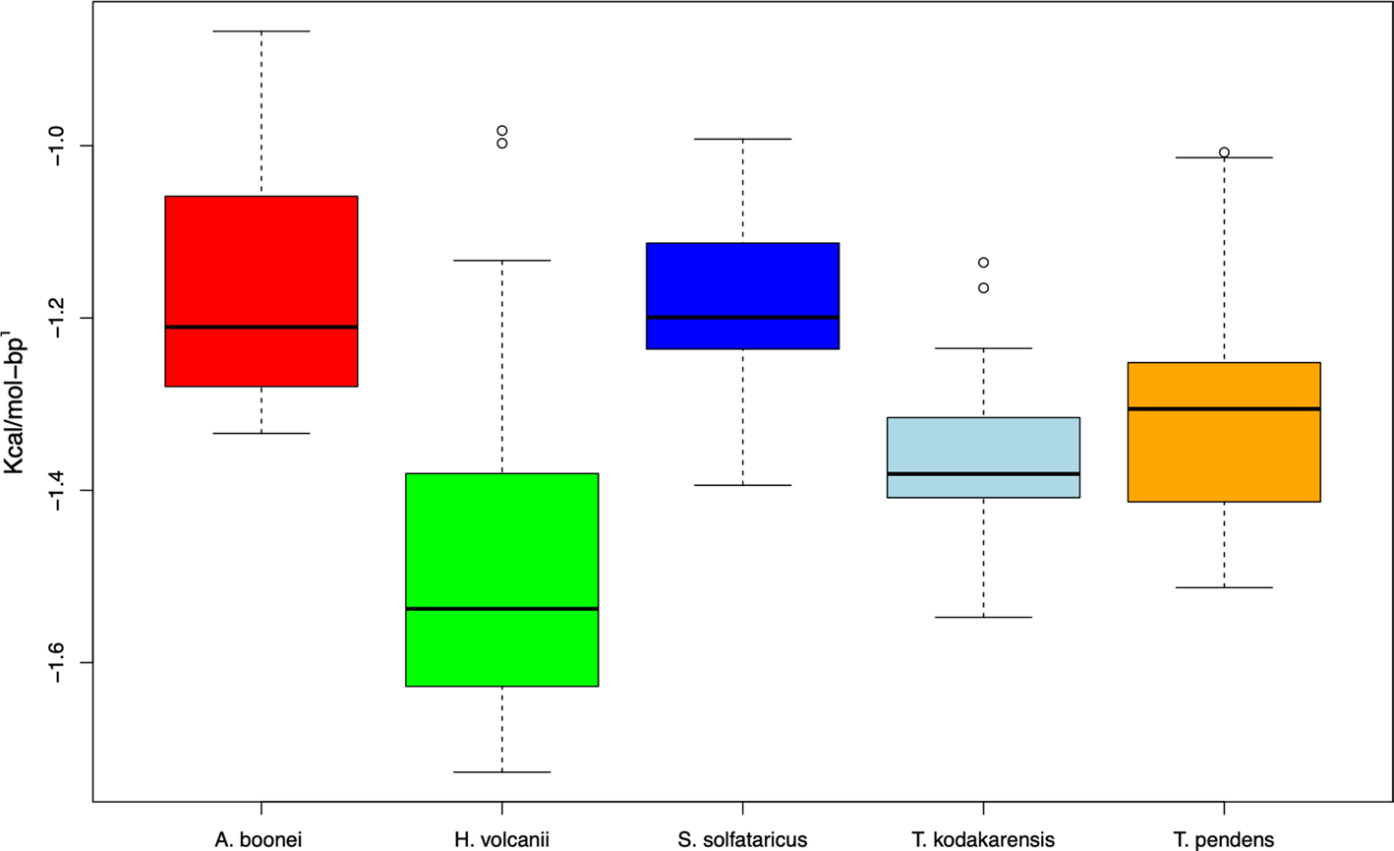
Boxplots of promoter sequences of five different archaea. Promoter sequences from five archaea were converted into DDS. Each position (− 80 to 0) had its value averaged to produce the boxplots. *H. volcanii* has been placed apart from the other organisms due to its higher GC content. Groups of promoters consisting of *A. boonei* and *S. solfataricus* and *T. pendens* and *T. kodakarensis* were observed

To validate the newly identified promoters, they have been compared with experimentally verified promoters. In this sense, Fig. 3 holds information of the DDS profile of *A. boonei* and *T. pendens* as well as the other three archaea. In Fig. 4, there is a conserved region in the binding site of TBP, TFB and TFE proteins for all observations. A statistical analysis of the slice − 40 to − 1 of Fig. 2 was provided in Fig. 5, from which unannotated promoters of *A. boonei* resemble the averages of *S. solfataricus*, while *T. pendens* match *T. kodakarensis*. The whole analysis of the datasets present a *p* = 3.241^ − 14.

The method proposed in this study was able to hand in regulatory annotation upon the genomes of *A. boonei* and *T. pendens*. To do so, we systematically characterized promoters from well-known archaea [29] and systematically used the algorithmized information in order to locate promoters in unannotated upstream regions of these organisms.

Many factors such as the diversity of archaea and their relatively recent discovery creates the need for high quality genome annotation. This is the moment when *in-silico* approaches provide help to experimental biology by curating data [20]. The boxplots portrayed in Fig. 5 showed two groups of organisms. No taxonomic inferences, i.e., boxplots with similar averages, could be made upon these since *T. kodakarensis* and *A. boonei* are Euryarchaea while *S. solfataricus* and *T. pendens* belong to the Crenarchaeota division, the statistical resemblance of these organisms requires further analysis. We also suggest using the model of *H. volcanii* in order to locate promoters in archaea that have high GC content. The statistical similarity found between verified and potential promoters advocate the robustness of the method proposed.

## Concluding remarks

The results gathered in this study reveal the classification of promoter sequences in archaea susceptible to the percentage of GC in specific organisms. Moreover, the classification indicates a novel way of predicting promoter sequences in unannotated archaeal genomes through a combination of artificial neural networks and statistics. In this regard, the structural parametrization of genetic information has been able to locate key areas within upstream regions, successfully classified in *A. boonei* and *T. pendens*.

## Supplementary Information

**Additional file 1: Table S1.** The following table contains the performance metrics of the ANN classificatory approach of three archaea with the three forms of control implemented in this study. The three controls have been averaged to present a single value/metric.

## Data Availability

All data employed in this study was made available in public repositories prior to its submission. The promoter sequences employed as well in this study as well as their annotation are publicly available at 10.5281/zenodo.5137551. In addition, the byproduct annotated sequences of the classification method of the is study is available at https://doi.org/10.5281/zenodo.5729308.

## References

[1] DeLong EF, Wu KY, Prézelin BB, Jovine RVM (1994). High abundance of Archaea in Antarctic marine picoplankton. Nature.

[2] Baker BJ, De Anda V, Seitz KW, Dombrowski N, Santoro AE, Lloyd KG (2020). Diversity, ecology and evolution of Archaea. Nat Microbiol.

[3] Coulson RMR, Touboul N, Ouzounis CA (2007). Lineage-specific partitions in archaeal transcription. Archaea.

[4] Leigh JA, Albers SV, Atomi H, Allers T (2011). Model organisms for genetics in the domain Archaea: Methanogens, halophiles, Thermococcales and Sulfolobales. FEMS Microbiol Rev.

[5] Werner F (2007). Structure and function of archaeal RNA polymerases. Mol Microbiol.

[6] Eme L, Spang A, Lombard J, Stairs CW, Ettema TJG (2017). Archaea and the origin of eukaryotes. Nat Rev Microbiol.

[7] Smollett K, Blombach F, Fouqueau T, Werner F, Clouet-d'Orval B (2017). A global characterisation of the Archaeal transcription machinery. RNA metabolism and Gene Expression in Archaea.

[8] Fouqueau T, Blombach F, Cackett G, Carty AE, Matelska DM, Ofer S, Pilotto S, Phung DK, Werner F (2018). The cutting edge of archaeal transcription. Emerg Top Life Sci.

[9] Martinez-Pastor M, Tonner PD, Darnell CL, Schmid AK (2017). Transcriptional regulation in Archaea: from individual genes to global regulatory networks. Annu Rev Genet.

[10] Soppa J (1999). Transcription initiation in Archaea: facts, factors and future aspects. Mol Microbiol.

[11] Haberle V, Stark A (2018). Eukaryotic core promoters and the functional basis of transcription initiation. Nat Rev Mol Cell Biol.

[12] Kadonaga JT (2012). Perspectives on the RNA polymerase II core promoter. Wiley Interdiscipl Rev Dev Biol.

[13] Babski J, Haas KA, Näther-Schindler D, Pfeiffer F, Förstner KU, Hammelmann M, Hilker R, Becker A, Sharma CM, Marchfelder A, Soppa J (2016). Genome-wide identification of transcriptional start sites in the haloarchaeon Haloferax volcanii based on differential RNA-Seq (dRNA-Seq). BMC Genom.

[14] She Q, Singh RK, Confalonieri F, Zivanovic Y, Allard G, Awayez MJ, Christina CY, Clausen IG, Curtis BA, De Moors A, Erauso G, Van Der Oostg J. The complete genome of the crenarchaeon Sulfolobus solfataricus P2. Proceedings of the national academy of sciences of the United States of America, 2001.10.1073/pnas.141222098 10.1073/pnas.141222098 PMC35428 11427726

[15] Jäger D, Förstner KU, Sharma CM, Santangelo TJ, Reeve JN (2014). Primary transcriptome map of the hyperthermophilic archaeon Thermococcus kodakarensis. BMC Genom.

[16] Bartlett MS, Thomm M, Geiduschek EP (2000). The orientation of DNA in an archaeal transcription initiation complex. Nat Struct Biol.

[17] Oubounyt M, Louadi Z, Tayara H, To Chong K (2019). Deepromoter: robust promoter predictor using deep learning. Front Genet.

[18] Ryasik A, Orlov M, Zykova E, Ermak T, Sorokin A (2018). Bacterial promoter prediction: selection of dynamic and static physical properties of DNA for reliable sequence classification. J Bioinform Comput Biol.

[19] Yella VR, Kumar A, Bansal M (2018). Identification of putative promoters in 48 eukaryotic genomes on the basis of DNA free energy. Sci Rep.

[20] Martinez GS, de Ávila e Silva S, Kumar A, Pérez-Rueda E (2021). DNA structural and physical properties reveal peculiarities in promoter sequences of the bacterium Escherichia coli K-12. SN Appl Sci.

[21] SantaLucia J, Hicks D (2004). The Thermodynamics of DNA structural motifs. Annu Rev Biophys Biomol Struct.

[22] Kanhere A, Bansal M (2005). Structural properties of promoters: Similarities and differences between prokaryotes and eukaryotes. Nucleic Acids Res.

[23] de Avila e Silva S, Echeverrigaray S, Gerhardt GJL (2011). BacPP: bacterial promoter prediction-a tool for accurate sigma-factor specific assignment in enterobacteria. J Theor Biol.

[24] Stone M (1974). Cross-Validatory choice and assessment of statistical predictions. J Roy Stat Soc Ser B.

[25] Beck MW (2018). NeuralNetTools: visualization and analysis tools for neural networks. J Stat Soft.

[26] Liu X, Guo Z, He T, Ren M (2020). Prediction and analysis of prokaryotic promoters based on sequence features. BioSystems.

[27] Geman S, Bienenstock E, Doursat R (1992). Neural networks and the bias/variance dilemma. Neural Comput.

[28] Afaq S, Rao S (2020). Significance of epochs on training a neural network. Int J Sci Technol Res.

[29] Martinez GS, Sarkar S, Kumar A, Pérez-Rueda E, de Avila e Silva S (2021). Characterization of promoters in archaeal genomes based on DNA structural parameters. MicrobiologyOpen.

[30] Hanzelka BL, Darcy TJ, Reeve JN (2001). TFE, an archaeal transcription factor in methanobacterium thermoautotrophicum related to eucaryal transcription factor TFIIEα. J Bacteriol.

[31] Takemasa R, Yokooji Y, Yamatsu A, Atomi H, Imanaka T (2011). Thermococcus kodakarensis as a host for gene expression and protein secretion. Appl Environ Microbiol.

[32] Kumar P, Ambekar S, Kumar M, Roy S. Data mining - methods applications and systems, 2020. 10.5772/intechopen.87784

[33] Mangal R, Nori AV, Orso A. Robustness of neural networks: A probabilistic and practical approach. Proceedings - 2019 IEEE/ACM 41st international conference on software engineering: new ideas and emerging results, ICSE-NIER 2019. 10.1109/ICSE-NIER.2019.00032

[34] Xu Y, Wang XB, Ding J, Wu LY, Deng NY (2010). Lysine acetylation sites prediction using an ensemble of support vector machine classifiers. J Theor Biol.

