# Supplementary material for: Machine learning and statistics shape a novel path in archaeal promoter annotation
Source: BMC Bioinformatics. 2022 May 10;23:171. doi: 10.1186/s12859-022-04714-x (PMC9087966; doi:10.1186/s12859-022-04714-x)
Supplement: Supplementary file 1 — Additional file 1: Table S1. The following table contains the performance metrics of the ANN classificatory approach of three archaea with the three forms of control implemented in this study. The three controls have been averaged to present a single value/metric. [file 12859_2022_4714_MOESM1_ESM.docx]

Additional file 1: Table S1—Average of three controls in the ANN method of classification

|  | Accuracy | Precision | Recall | Specificity |
| --- | --- | --- | --- | --- |
| *H. volcanii* | 89.37 | 89.52 | 89.25 | 89.55 |
| *S. solfataricus* | 87.67 | 87.64 | 87.83 | 87.61 |
| *T. kodakarensis* | 90.92 | 89.72 | 89.88 | 89.88 |
